# Molecular Characterization of Influenza Strains in Patients Admitted to Intensive Care Units during the 2017–2018 Season

**DOI:** 10.3390/ijms20112664

**Published:** 2019-05-30

**Authors:** Antonio Piralla, Elena Pariani, Federica Giardina, Cristina Galli, Davide Sapia, Laura Pellegrinelli, Federica Novazzi, Giovanni Anselmi, Francesca Rovida, Francesco Mojoli, Danilo Cereda, Sabrina Senatore, Fausto Baldanti

**Affiliations:** 1 Molecular Virology Unit, Microbiology and Virology Department, Fondazione IRCCS Policlinico San Matteo, 27100 Pavia, Italy; federica.giardina01@universitadipavia.it (F.G.); davide.sapia01@universitadipavia.it (D.S.); novazzifederica@gmail.com (F.N.); f.rovida@smatteo.pv.it (F.R.); f.baldanti@smatteo.pv.it (F.B.); 2 Department of Biomedical Sciences for Health, University of Milan, 20133 Milan, Italy; elena.pariani@unimi.it (E.P.); cristina.galli@unimi.it (C.G.); laura.pellegrinelli@unimi.it (L.P.); giovanni.anselmi@unimi.it (G.A.); 3 Department of Clinical, Surgical, Diagnostic and Pediatric Sciences, University of Pavia, 27100 Pavia, Italy; francesco.mojoli@unipv.it; 4 Anesthesia and Intensive Care, Emergency Department, Fondazione IRCCS Policlinico S. Matteo, 27100 Pavia, Italy; 5 DG Welfare, UO Prevenzione, Lombardy Region, 20124 Milan, Italy; Danilo_Cereda@regione.lombardia.it (D.C.); Sabrina_Senatore@regione.lombardia.it (S.S.)

**Keywords:** influenza virus, intensive care unit, severe respiratory infections, viral load, lower respiratory tract infection

## Abstract

This study aimed at assessing the frequency and the distribution of influenza virus types/subtypes in 172 laboratory-confirmed influenza-positive patients admitted to intensive care units (ICUs) during the 2017–2018 season in the Lombardy region (Northern Italy), and to investigate the presence of molecular pathogenicity markers. A total of 102/172 (59.3%) patients had influenza A infections (83 A/H1N1pdm09, 2 H3N2 and 17 were untyped), while the remaining 70/172 (40.7%) patients had influenza B infections. The 222G/N mutation in the hemagglutinin gene was identified in 33.3% (3/9) of A/H1N1pdm09 strains detected in the lower respiratory tract (LRT) samples and was also associated with more severe infections, whereas no peculiar mutations were observed for influenza B strains. A single-point evolution was observed in site 222 of A/H1N1pdm09 viruses, which might advantage viral evolution by favouring virus binding and replication in the lungs. Data from 17 paired upper respiratory tract (URT) and LRT samples showed that viral load in LRT samples was mostly higher than that detected in URT samples. Of note, influenza viruses were undetectable in 35% of paired URT samples. In conclusion, LRT samples appear to provide more accurate clinical information than URT samples, thus ensuring correct diagnosis and appropriate treatment of patients with severe respiratory infections requiring ICU admission.

## 1. Introduction

Influenza A and B viruses are transmitted efficiently from human to human and follow a seasonal epidemic pattern that in countries with temperate climates occurs mainly during winter [1]. Influenza A viruses (namely subtypes H1N1pdm09 and H3N2) and influenza B viruses (lineage B-Yagamata and B-Victoria) are currently the cause of seasonal epidemics [1]. 

Every year, seasonal influenza affects 5–10% of adults and 20–30% of children and is responsible for 3–5 million cases of severe illness and up to 650,000 deaths worldwide [2]. Although most influenza cases are mild, some patients develop severe acute respiratory infections (SARIs) and acute respiratory distress syndrome (ARDS), thus requiring admission to intensive care units (ICU). While underlying clinical conditions play an important role in the pathogenesis of severe respiratory syndromes, some viral strains (such as A/H1N1pdm09) appear to significantly and independently contribute to the development of SARI and ARDS [3]. Thus, since 2009, international health authorities have recommended the investigation of severe and complicated cases of influenza [4]. An annual seasonal influenza vaccination is recommended for pregnant women, the elderly, young children, immunocompromised people and people with chronic and cardiovascular disease who are at high risk of developing influenza complications [5].

Influenza A and B viruses are constantly evolving, particularly due to the pressure exerted by the host’s immune system that results in the antigenic drift of viral surface protein hemagglutinin (HA) and neuraminidase (NA) [6]. Genetic variations in the viral genome may increase infectivity or pathogenicity of the virus and lead to expanded tissue tropism, which can worsen clinical outcomes and cause antiviral drug resistance [7,8,9,10]. Moreover, antigenic shifts may occur in influenza A viruses, as occurred 10 years ago with the emergence of the swine-origin A/H1N1pdm09 influenza virus, which was considered a major pandemic threat to human health [11]. As each influenza season is characterised by specific patterns of circulating influenza viruses, the identification and characterisation of influenza viruses is essential in order to develop effective vaccines against the influenza strains predicted to circulate in the upcoming season.

The aims of this study were (i) to assess the frequency and the distribution of influenza virus types and subtypes in patients admitted to ICUs during the 2017–2018 influenza season in the Lombardy region (10 million inhabitants) in northern Italy; (ii) to carry out molecular and phylogenetic analyses of the HA gene sequences of influenza A/H1N1pdm09, A/H3N2 and B viruses; (iii) to determine the epidemiological and molecular characteristics of influenza viruses in severe and mild respiratory infections in order to identify molecular pathogenicity markers. 

## 2. Results

### 2.1. Laboratory-Confirmed Influenza ICU Cases

**Figure 1:**
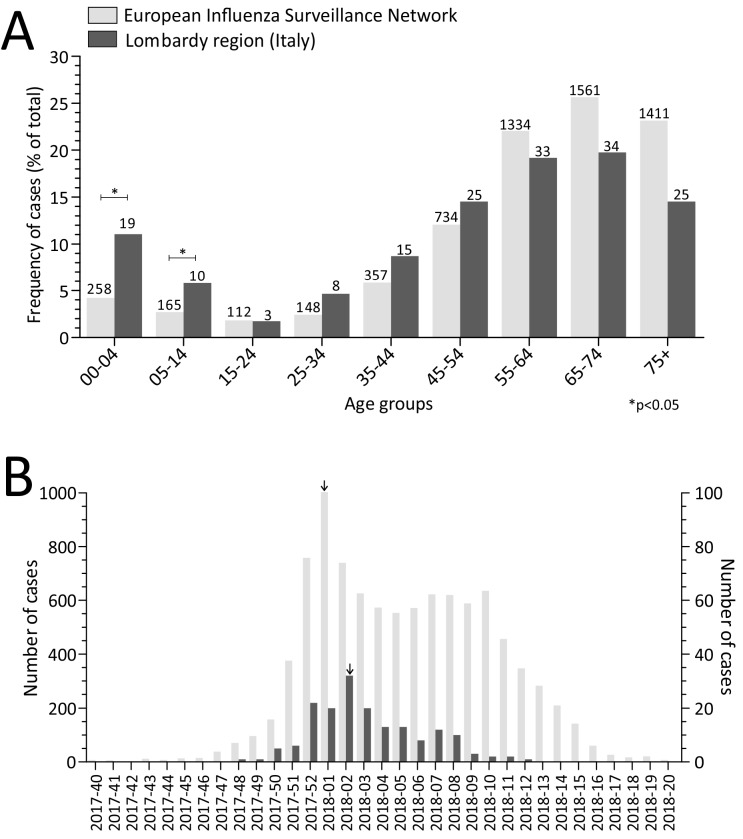
Laboratory-confirmed influenza in intensive care unit (ICU)-admitted cases by age group (**A**) and by week (**B**) observed in the framework of the European Influenza Surveillance Network (EISN) [12] and in Lombardy. Number of patients is reported on the top of each column. The peak of cases is reported with an arrow above the column.

A total of 172 laboratory-confirmed influenza cases were observed among patients admitted to ICUs in the Lombardy region during the 2017–2018 season. The median age of these patients was 50 years old (ranging from 1 month old to 86 years old) with 88 (51.2%) being males. The majority (143/172; 83.1%) of the influenza-associated ICU patients were adults (>15 years old; Figure 1A). The percentage of paediatric patients admitted to the ICUs was higher than the percentage reported by the European Influenza Surveillance Network (EISN) [12] (16.9% vs 6.9%; Figure 1A). The majority (131/172, 76.2%) of patients had at least one comorbidity (i.e., cardiovascular diseases, chronic respiratory diseases, diabetes, cancer). Five severe influenza cases occurred in pregnant women (second/third trimester). One hundred (58.1%) patients required mechanical ventilation for respiratory failure and 22/100 (22.0%) of these patients required extracorporeal membrane oxygenation (ECMO) support. Death occurred in 13.4% (23/172) of all cases. All apart from one of the fatal cases occurred in individuals older than 52 years and 87.0% (20/23) of whom had pre-existing medical conditions. Treatment with oseltamivir was initiated in 143 (83.0%) patients. The majority (73.6%, 67/91 of severe cases occurred in patients that had not been vaccinated (information on the 2017–2018 vaccination status was available for 91/172 of the cases). 

A total of 102/172 (59.3%) patients had influenza A infections while the remaining 70/172 (40.7%) patients had influenza B infections. Eighty-three out of 102 (81.4%) influenza A cases were typed as influenza A/H1N1pdm09, 2/102 (1.9%) as influenza A/H3N2 while the remaining 17/102 (16.7%) cases could not be typed because of low viral load. 

### 2.2. Temporal Distribution of Severe Influenza Cases

In the first 10 weeks of surveillance (from week 42, 2017 to week 51, 2017), there were few severe cases (<10% of the total; Figure 1B). The 2017–2018 influenza season reached its peak in week 2/2018 with 32 cases (18.2% of the total). Following the peak, the number of cases decreased but remained roughly constant (approximately 10 cases per week) for at least 6 weeks (from week 3, 2018 to week 8, 2018). Finally, only five cases were observed from week 9, 2018 to week 17, 2018. 

### 2.3. Phylogenetic Analyses

HA sequencing was carried out for 58/83 (69.9%) influenza A/H1N1pdm09 and 33/70 (47.1%) influenza B strains. There were 49/58 (84.5%) influenza A/H1N1pdm09 strains identified in upper respiratory tract (URT) samples, while 9/58 (15.5%) were detected in lower respiratory tract (LRT) samples. There were 28/33 (84.8%) influenza B strains identified in the URT samples, while 5/33 (15.2%) were detected in the LRT samples. A total of 27 influenza A/H1N1pdm09 and 59 influenza B strains obtained from unhospitalized patients with mild respiratory infection were included in the phylogenetic analyses as controls. 

**Figure 2:**
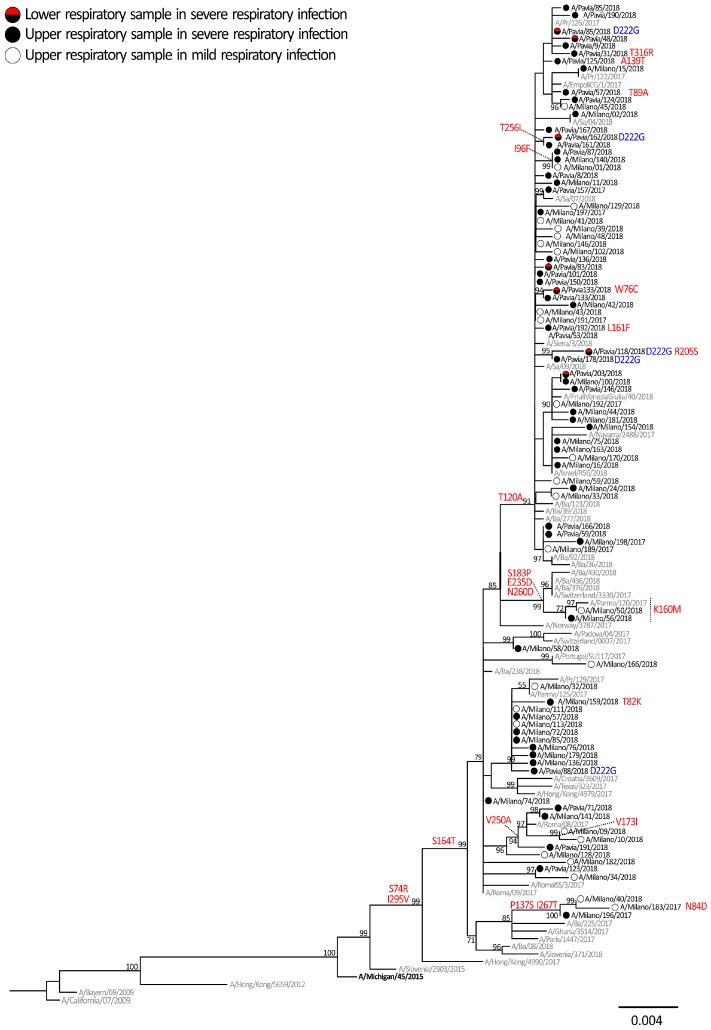
Phylogenetic tree of hemagglutinin (HA) sequences of A/H1N1pdm09 viruses, which was inferred by using the maximum likelihood. The percentage of replicate trees in which the associated taxa clustered together in the bootstrap test (1000 replicates) is shown next to the branches. A/H1N1pdm09 sequences originally identified in this study are reported with black circles (upper respiratory tract (URT) sample of severe cases), red/black circles (lower respiratory tract (LRT) samples of severe cases) and white circles (URT samples of mild cases). The 2017–2018 A/H1N1pdm09 vaccine strain is reported in bold and reference strains are reported in grey. Amino acid mutations are reported in red, mutations at codon 222 are in blue.

The phylogenetic analysis showed that all influenza A/H1N1pdm09 HA sequences belonged to the 6B genetic group, genetic subgroup 6B.1, represented by the A/Michigan/45/2015 reference strain, which was included in the 2017–2018 vaccine (Figure 2). The overall identity between the influenza A/H1N1pdm09 strains that circulated in Lombardy and the vaccine strain A/Michigan/45/2015 included in the 2017–2018 and 2018–2019 seasonal influenza vaccines ranged from 98.2% to 99.1%. All influenza A/H1N1pdm09 strains were characterized by the S74R, S164T and I295V amino acid substitutions, also observed in most A/H1N1pdm09 strains, that circulated in the northern hemisphere late in the 2017–2018 season. Most (42/58, 72.4%) strains identified in patients with severe infections and about half (15/27, 55.6%) of strains observed in patients with mild influenza-like illness (ILI) belonged to a genetic subgroup distinguished by amino acid substitution T120A. Other subgroups characterized by specific amino acid substitutions were observed for a smaller number of strains (i.e., S183P, E235D and N260D). 

**Figure 3:**
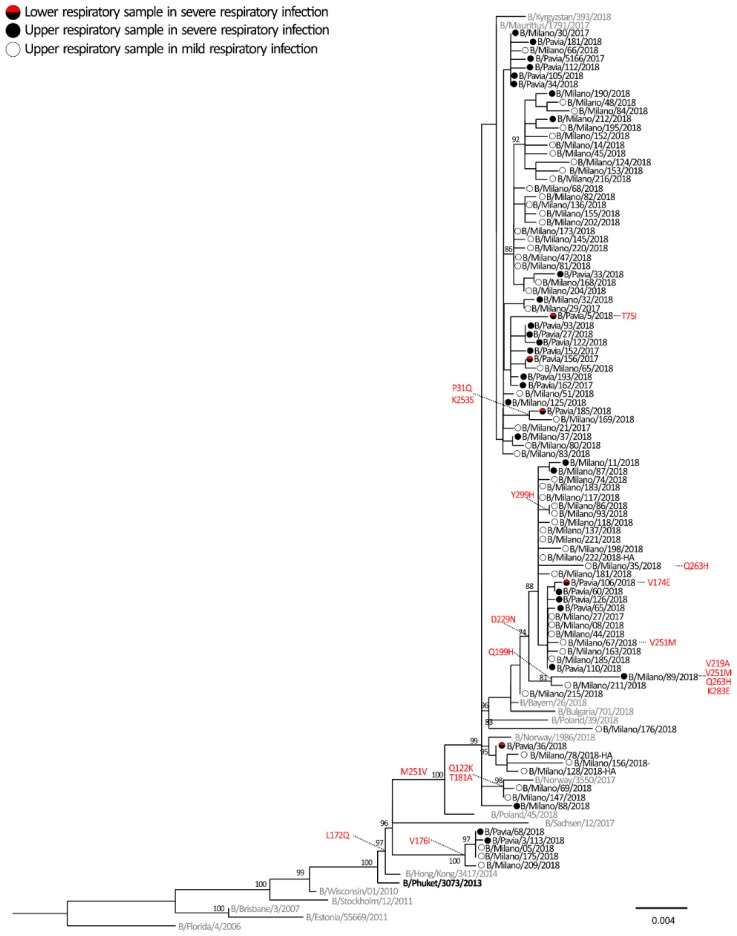
Phylogenetic tree of HA sequences of B viruses which was inferred by using the maximum likelihood. The percentage of replicate trees in which the associated taxa clustered together in the bootstrap test (1000 replicates) is shown next to the branches. Influenza B sequences originally identified in this study are reported with black circles (URT sample of severe case), red/black circles (LRT samples of severe cases) and white circles (URT samples of mild cases). The vaccine strain is reported in bold and reference strains are reported in grey. Amino acid mutations are reported in red.

All HA sequences of influenza B viruses belonged to the Yamagata lineage and, in particular to clade 3, were represented by the reference strain B/Phuket/3073/2013 included in the 2017–2018 quadrivalent vaccine but not in the trivalent vaccine (Figure 3). All of the 91 strains analysed belonged to a clade 3 subgroup and were characterized by two amino acid substitutions (L172Q and M251V) with respect to the vaccine strain. These mutations were identified in most of the B/Yamagata strains circulating during the 2017–2018 season in the northern hemisphere. Other minor subgroups were observed among the B/Yamagata sequences and one of these clusters was characterized by amino acid substitution D229N. A single strain, B/Milan/89/2018, identified in a patient with a severe infection, had undergone a series of peculiar mutations: Q199H, V219A, V251M, Q263H and K283E (Figure 3).

### 2.4. Molecular Signatures of Increased Virulence in HA Gene of Influenza virus A and B 

HA sequences from ICU patients were also analysed in order to find mutations potentially associated with greater clinical severity. The analyses carried out on influenza A/H1N1pdm09 and B strains focused on critical amino acids involved in the interaction with cell receptors and their surrounding genomic region. Mutations at codon 222 were observed in 5/58 (8.6%) of influenza A/H1N1pdm09 (D222G/N/A) strains as shown in blue in Figure 2. However, the frequency of 222 mutation was higher (33.3%, 3/9) when considering only the influenza A/H1N1pdm09 strains detected in the LRT samples (*n* = 9). The remaining two (4.1%, 2/49) mutated strains were detected in the URT samples (Figure 2). No changes in position 222 were observed in the strains identified in mild influenza cases. Among influenza B strains, only two T75I and V174E changes were identified in LRT samples of two severe cases, B/Pavia/05/2018 and B/Pavia/106/2018, respectively. No other changes were exclusively observed in influenza B strains of severe respiratory cases. 

### 2.5. Selective Pressure Analyses

The selective pressures acting on the HA segment were assessed for the alignment of influenza A and B HA sequences, the results of which are summarized in Table 1. A global analysis of selective pressure, performed using the single-likelihood ancestor (SLAC) model, indicated an estimated overall mean number of non-synonymous changes per non-synonymous site (dN)/number of synonymous changes per synonymous site (dS) ratio of 0.18 for influenza A and 0.10 for influenza B. Our analyses identified two sites (positions 137 and 222) in influenza A/H1N1pdm09 alignment and no sites in the influenza B virus as being under positive selection with at least two of the methods used. Contrastingly, several sites were identified under neutral and purifying selective pressures. It is important to note that both positive selected sites in the influenza A/H1N1pdm09 strains were included in the antigenic sites, and in particular, position 222 has been proven to be essential for recognition of receptors.

### 2.6. Viral Load in Paired URT and LRT Samples

**Figure 4:**
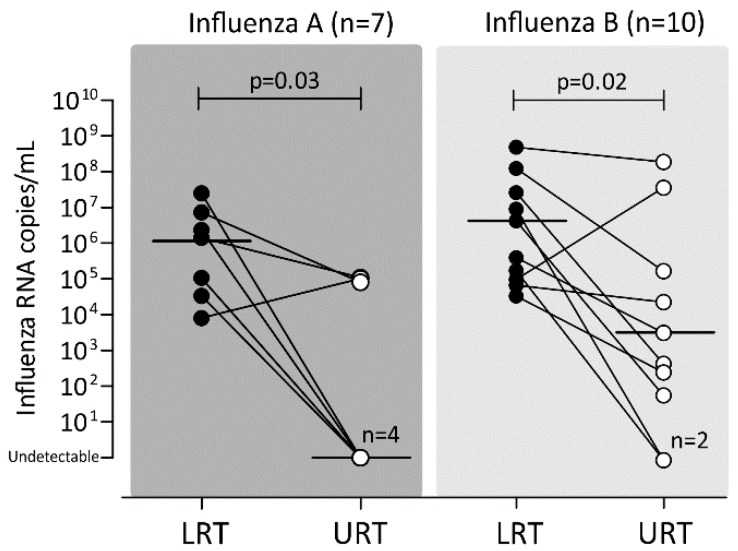
Comparison of viral loads detected in paired URT and LRT samples in patients diagnosed with influenza A and B. Upper respiratory tract, URT; lower respiratory tract; LRT.

Paired URT and LRT samples were available from 17 patients (7 influenza A virus and 10 influenza B virus infections). The viral load in the LRT samples was higher than that detected in the URT samples for almost all influenza A (6/7, 85.7%) and influenza B (9/10, 90.0%) cases. The median viral load was significantly higher in LRT secretions than in URT for both influenza A (*p* = 0.03) and B (*p* = 0.02) cases (Figure 4). It is important to note that the URT samples were undetectable in 4/7 (57.1%) influenza A cases and 2/10 (20.0%) influenza B cases. 

## 3. Discussion

During the 2017–2018 influenza season, the majority of influenza viruses detected in Europe were of type B, accounting for nearly 63% of all positive samples, while the remaining 37% of the cases were infected with influenza A [13]. However, SARI surveillance showed slightly different results for the European epidemiological scenario, since influenza A viruses proved to be the most commonly detected. More specifically, approximately 53% of the over 9000 laboratory-confirmed severe cases were caused by type A viruses, while 47% were influenza B virus infections [13]. In Italy, a total of 764 laboratory-confirmed influenza cases requiring ICU admission were reported during the 2017–2018 epidemic, with nearly 23% (*n* = 172) of these cases occurring in Lombardy [14]. 

Virological surveillance of severe influenza cases foresaw the molecular characterization of the HA gene of the circulating strains. The influenza A/H1N1pdm09 strains detected in severe cases were identical to those belonging to subgroup 6B.1, which were detected in mild cases. A similar trend was observed for influenza B strains—the circulating strains were phylogenetically and antigenically similar to the B/Yamagata vaccine strain of the 2017–2018 and 2018–2019 seasons (B/Phuket/3073/2013) [15]. Overall, the molecular characterization of the HA gene showed that the influenza strains detected in severe influenza cases were molecularly similar to those frequently detected in mild influenza cases. Few amino acid changes became fixed in the population of circulating viruses, in fact, some subgroups grouped separately in the phylogenetic trees. However, these changes did not cause significant antigenic modifications in influenza A/H1N1pdm09 and B strains [16]. 

Some host risk factors, namely pregnancy and obesity, were previously associated with more severe influenza A/H1N1pdm09 infections [17]. One of the objectives of this study was to investigate the presence of mutations associated with increased clinical severity. More specifically only few genetic modifications have been associated with increased transmissibility, replicative efficiency and tissue tropism range during the A/H1N1 pandemic in 2009 [7,8,18]. It has been demonstrated that a single amino acid change (222G/N) in the hemagglutinin gene of influenza A/H1N1pdm09 may lead to an increase in viral replication in the lower respiratory tract and a worsening of clinical conditions [19,20,21]. Among the A/H1N1pdm09 strains analysed in this study, 8% had mutations at position 222 (G/N/A) but the percentage was lower when considering only URT samples. The mutation frequency of 222 increased to approximately 30% when only LRT samples were considered. These findings were in line with our previous observations for the 2009–2017 influenza seasons [7,22,23,24]. Finally, the presence of the 222G variant in upper respiratory tract samples may suggest the possible inter-human transmission of this aggressive variant. However, further studies are required to evaluate the fitness of these variants in the context of upper respiratory tract infection. 

Influenza B infections are generally deemed to be less severe and less threatening to public health than influenza A viruses; however, it has been reported that the influenza B virus can sometimes cause encephalitis, myositis and even death [25]. However, relatively few studies on mutations associated with increased disease severity of influenza B infections have been published. In this study, amino acid changes in antigenic sites or receptor binding sites were investigated. Only two unique mutations (T75I and V174E) were observed in these sites, suggesting the absence of specific genetic markers for influenza B viruses. Therefore, the increased number of influenza B cases observed in ICU patients during the 2017–2018 season may be due to the high level of circulation of influenza B viruses in the general population. 

Our data on overall dN/dS rate are in line with previous observations suggesting a strong purifying selection pressure on the HA gene of influenza A and B strains [26]. However, two sites of influenza A/H1N1pdm09 were found to be under positive selection by at least two methods, one of which was position 222. This positively selected site in position 222 in the HA of the H1N1pdm09 virus is probably due to the molecular adaptation of the virus, which may confer an evolutionary advantage for the virus. However, positive selection appears to be a rare event, as it only occurs at few amino acid sites in a short period of time [27]. Moreover, it is hard to detect positive sites when compared against a large number of sites under neutral and purifying selection pressures, as observed in our datasets [27].

Data from paired URT/LRT samples showed that the virus was undetectable in URT sample of 35% of cases. Our data are in line with those obtained from previous studies [28,29] and confirm our previous observations [7,22,24]. From a general perspective, in patients with severe respiratory illnesses in absence of viral detection in URT samples, empirical antiviral treatment and LRT sample analyses should be considered in order to improve patient management and diagnosis [30,31]. Clinicians should be aware that sampling of the upper airways may not accurately diagnose the infection.

## 4. Material and Methods

### 4.1. Study Design

From 16 October 2017 (week 42, 2017) to 29 April 2018 (week 17, 2018), respiratory samples were prospectively collected from SARI patients requiring ICU admission and from outpatients with ILI by sentinel general practitioners and analysed in two reference laboratories (Molecular Virology Unit, Fondazione IRCCS Policlinico San Matteo, Pavia, and Department of Biomedical Sciences for Health, University of Milan) as part of the Regional Influenza Surveillance Plan in the Lombardy region (10 million inhabitants). Whenever possible, paired URT (i.e., nasal swabs and nasopharyngeal aspirates) and LRT (i.e., bronchoalveolar lavages or broncho-aspirates) samples were obtained from SARI patients. SARI is defined as an acute respiratory infection with a history of fever or measured fever of >38 °C and cough, with onset within the last 10 days, requiring hospitalization [32]. ILI is defined as a sudden onset of fever (>38 °C) or feverishness with one or more respiratory symptoms (cough, sore throat and/or shortness of breath) and one or more systemic symptoms (myalgia, headache and/or malaise) [32]. 

### 4.2. Ethical Statement

This study was performed according to the guidelines of the Institutional Review Board on the use of biological specimens for scientific purposes in keeping with Italian law (art.13 D.Lgs 196/2003). Informed consent for influenza detection, typing and molecular characterization was not required since patients with SARI and ILI were included in the regional diagnostic and clinical management protocol. Data from hospitalized patients and ILI cases were analysed anonymously in accordance with the National Surveillance Plan (InfluNet).

### 4.3. Detection and Molecular Characterization of Influenza Viruses

Respiratory samples were extracted using a QIAsymphony instrument with QIAsymphony DSP Virus/Pathogen Midi Kit (Complex 400 protocol) according to the manufacturer’s instructions (Qiagen, Hilden, Germany). A panel of real-time (RT)-polymerase chain reactions (PCR) was performed to detect and quantify respiratory viruses, including influenza A (with subtyping A/H1N1pdm09 and A/H3N2) and B [33,34]. 

### 4.4. Sequencing of Influenza HA Gene

The HA gene of influenza A/H1N1pdm09 and influenza B viruses was amplified directly from clinical specimens using the SuperScriptIII One-Step RT-PCR amplification kit (Invitrogen, Carlsbad, CA, USA) and specific primers [24,35]. Purified PCR products were sequenced using the BigDye Terminator Cycle-Sequencing kit (Applied Biosystems, Foster City, CA, USA) in an ABI Prism 3130xl Genetic Analyzer (Applied Biosystems, Foster City, CA, USA).

### 4.5. Phylogenetic Analyses

The sequences were assembled using Sequencher software, version 4.6 (Gene Codes Corporation, Ann Arbor, MI, USA). Nucleotide alignments were constructed using the ClustalW program embedded in MEGA version 7 software [36]. A maximum likelihood phylogenetic tree was inferred using the IQ-Tree web server (version 1.6.8) [37] and the robustness of branches was evaluated using the ultrafast bootstrap approximation tests with 1000 replicates. The nucleotide sequences identified in this study have been submitted to the GenBank database with accession numbers MK829607-MK829639, MK829789-MK829811, MK949213-MK949280 and MK949286-MK949339.

### 4.6. Selective Pressure 

Tests for positive selection were conducted using single-likelihood ancestor counting (SLAC), fixed-effects likelihood (FEL), the mixed effects model of evolution (MEME) and fast unconstrained Bayesian approximation (FUBAR) on the Datamonkey server [38] and the dN/dS ratios were calculated using the SLAC and FEL codon-based maximum likelihood approaches. SLAC counts the number of non-synonymous changes per non-synonymous site (dN) and tests whether it is significantly different from the number of synonymous changes per synonymous site (dS). FEL estimates the ratios of non-synonymous to synonymous changes for each site in an alignment [39]. In order to avoid an excessive false-positive rate, sites with SLAC, FEL and MEME *p*-values <0.1 and a FUBAR posterior probability >0.90 were accepted as candidates for selection.

### 4.7. Statistical Analysis 

Comparisons of viral loads in paired respiratory samples were performed with the Wilcoxon rank sum test for continuous paired variables. Fisher’s exact test for categorical variables was used for analysing mutation frequencies between groups of patients. All of the statistical analyses were performed using Graph Pad Prism software (version 5.00.288).

## 5. Conclusions

During the 2017–2018 influenza season, the sustained circulation of influenza B viruses (approximately 63%) increased the number of patients admitted to ICUs diagnosed with influenza B infections, while the number of cases caused by influenza A/H1N1pdm09 strains remained constant compared with other seasons. A single point evolution was observed in the site 222 of A/H1N1pdm09 viruses, which may have contributed to the evolutionary advantage of the virus by favouring virus attachment and replication in the lung. Finally, lower respiratory tract samples provided more valid clinical information than upper respiratory samples, thus ensuring the correct diagnosis and appropriate treatment of patients diagnosed with severe respiratory infections requiring ICU admission. 

## Figures and Tables

**Table 1:** Positive and negative selected sites for the influenza A/H1N1pdm09 and influenza B strains identified in this study.

Methods	Influenza A/H1N1pdm09 Virus Codon		Influenza B Virus Codon	
	Positive	Negative	Positive	Negative
SLAC	None	431, 472, 544	None	19 sites
FEL	120, 137, 205, 250	472, 544	122, 181	None
FUBAR	137, 222	39 sites	229, 251	114 sites
MEME	222	None	253	None

SLAC: single-likelihood ancestor; FEL: fixed-effects likelihood; FUBAR: fast unconstrained Bayesian approximation; MEME: mixed effects model evolution.
